# Hepatic Metabolic Dysregulation as a Potential Amplifier of Leukemogenesis Following mRNA Vaccination: A Novel Mechanistic Hypothesis

**DOI:** 10.3390/medicina61091687

**Published:** 2025-09-17

**Authors:** Batuhan Erdoğdu, Ozan Kaplan, Mustafa Çelebier, Ümit Yavuz Malkan, İbrahim Celalettin Haznedaroğlu

**Affiliations:** 1 Department of Hematology, Faculty of Medicine, Gülhane Training and Research Hospital, 06010 Ankara, Türkiye; 2 Department of Analytical Chemistry, Faculty of Pharmacy, Hacettepe University, 06230 Ankara, Türkiye; ozantiger@gmail.com (O.K.); celebier@hacettepe.edu.tr (M.Ç.); 3 Department of Hematology, Faculty of Medicine, Hacettepe University, 06230 Ankara, Türkiye; umitmalkan@hotmail.com (Ü.Y.M.); ichaznedaroglu@gmail.com (İ.C.H.)

**Keywords:** leukemogenesis, mRNA vaccines, hepatic metabolism, metabolic crosstalk, folate metabolism, tryptophan catabolism, iron homeostasis, NADPH metabolism

## Abstract

*Background:* The liver’s role as a metabolic gatekeeper positions it uniquely to influence systemic metabolic homeostasis and potentially modulate leukemogenesis through hepato-hematopoietic crosstalk. Recent observations of rare hematological malignancies following mRNA vaccination warrant mechanistic investigation. *Hypothesis:* We propose that mRNA vaccines, through their preferential hepatic tropism via lipid nanoparticles (LNPs), may transiently dysregulate hepatic metabolism in susceptible individuals, creating metabolic perturbations that amplify pre-existing leukemogenic vulnerabilities through five interconnected mechanisms: (1) competitive folate sequestration for vaccine-induced lymphoproliferation, potentially starving bone marrow precursors of essential one-carbon units; (2) hepatic lipid processing overload from LNP accumulation, exacerbating phospholipid dysregulation in pre-leukemic clones; (3) cytokine-mediated upregulation of hepatic indoleamine 2,3-dioxygenase (IDO), accelerating tryptophan catabolism and creating an immunosuppressive milieu favoring leukemic escape; (4) inflammatory induction of hepcidin, sequestering hepatic iron while triggering compensatory intestinal iron hyperabsorption and potential bone marrow iron overload; and (5) increased hepatic NADPH demand for antioxidant defense and lipid metabolism, diverting reducing equivalents from bone marrow stromal support. *Implications:* This hypothesis suggests that transient hepatic metabolic perturbations may create a permissive milieu for leukemogenesis in metabolically vulnerable individuals. The proposed mechanisms generate testable predictions and identify potential therapeutic targets, including folate supplementation, IDO inhibition, and iron chelation in high-risk cohorts.

## 1. Introduction

Leukemogenesis represents a complex multistep process involving the accumulation of genetic and epigenetic alterations within hematopoietic stem and progenitor cells, ultimately leading to malignant transformation and clonal expansion [1,2]. While the genetic underpinnings of leukemia have been extensively characterized, the role of metabolic perturbations in facilitating or accelerating leukemogenic processes remains incompletely understood. Emerging evidence suggests that metabolic reprogramming is not merely a consequence of oncogenic transformation but may actively contribute to the initiation and progression of hematological malignancies [3,4,5].

The advent of mRNA vaccine technology has revolutionized immunization strategies, particularly during the COVID-19 pandemic. These vaccines utilize lipid nanoparticles (LNPs) as delivery vehicles, which demonstrate pronounced hepatic tropism due to apolipoprotein E-mediated uptake via hepatic low-density lipoprotein receptors [6]. Preclinical and regulatory studies indicate that LNP-formulated mRNA vaccines preferentially accumulate in the liver, leading to significant antigen expression and potential transient metabolic perturbations [7]. Although vaccine-associated liver injury is rare, these findings highlight the liver as a central mediator of systemic metabolic changes that could influence hematopoietic homeostasis in susceptible individuals [8].

While the safety profile of mRNA vaccines has been extensively documented, rare reports of hematological malignancies following vaccination have emerged, including a case of B-cell lymphoblastic lymphoma in a murine model following intravenous BNT162b2 administration [9]. Such observations, while not establishing causality, warrant mechanistic investigation into potential pathways linking vaccination to leukemogenesis in susceptible individuals [10,11].

The liver functions as a metabolic gatekeeper, orchestrating systemic nutrient homeostasis and regulating the availability of metabolites essential for hematopoiesis [12]. Through its extensive portal circulation and specialized hepatocyte populations, the liver controls the systemic exposure to amino acids, lipids, vitamins, and trace elements that are critical for bone marrow function [13,14,15,16]. This hepato-hematopoietic axis represents a potential conduit through which hepatic metabolic perturbations could influence leukemogenic processes.

We propose a novel mechanistic hypothesis positioning the liver as a potential amplifier of vaccine-associated metabolic shifts that may contribute to leukemogenesis in metabolically susceptible individuals. This hypothesis posits that mRNA vaccines, through their preferential hepatic accumulation and subsequent metabolic effects, may transiently alter the hepato-hematopoietic metabolic axis through five interconnected pathways involving folate utilization, lipid processing, tryptophan catabolism, iron regulation, and NADPH distribution. These transient perturbations may create a metabolic milieu that favors the expansion or progression of pre-leukemic clones in individuals harboring underlying metabolic vulnerabilities or genetic predispositions.

The clinical significance of this hypothesis extends beyond theoretical considerations, as it generates testable predictions and identifies potential therapeutic targets for both prevention and treatment. By elucidating these putative mechanisms, we aim to stimulate research into metabolic factors influencing leukemogenesis and identify novel interventional strategies targeting the hepato-hematopoietic metabolic crosstalk.

### 1.1. Mechanistic Framework and Biological Rationale

Hepatic Tropism of mRNA Vaccine Delivery Systems: The biodistribution profile of mRNA vaccines is fundamentally determined by the physicochemical properties of their lipid nanoparticle delivery systems. These formulations, typically comprising ionizable cationic lipids, phospholipids, cholesterol, and polyethylene glycol-conjugated lipids, demonstrate pronounced hepatic accumulation within minutes of systemic administration [6,17]. This hepatic tropism results from the rapid association of circulating LNPs with apolipoprotein E, facilitating recognition and uptake by hepatic low-density lipoprotein receptors. Consequently, the liver becomes the primary site of mRNA translation and antigen production, positioning it as a critical organ in vaccine-induced immune responses [6,18,19,20]. The metabolic consequences of this preferential hepatic accumulation remain incompletely characterized. LNPs may influence hepatic lipid homeostasis through direct interactions with cellular membranes and intracellular lipid processing machinery.

Importantly, this pattern of hepatic accumulation is not unique to mRNA vaccines but rather represents a broader pharmacological principle of nanoparticle-based therapeutics. For example, small interfering RNA (siRNA) therapies such as patisiran, formulated in lipid nanoparticles, also demonstrate preferential hepatic uptake due to apolipoprotein E–mediated endocytosis by hepatocytes and Kupffer cells [21,22]. Similarly, liposomal doxorubicin and other liposomal anticancer agents accumulate in the liver and spleen as part of the reticuloendothelial system’s clearance mechanisms [23]. In contrast, traditional oral small-molecule drugs undergo hepatic metabolism predominantly through first-pass clearance by cytochrome P450 enzymes, without significant nanoparticle-mediated accumulation [24]. On the other hand, monoclonal antibodies (mAbs) are generally not characterized by hepatic tropism, as they rely on target-mediated distribution and are primarily catabolized in peripheral tissues and the reticuloendothelial system rather than through hepatic uptake [25].

Furthermore, the immune activation resulting from hepatic antigen production may induce inflammatory cascades that alter hepatic metabolic function. Recent metabolomic analyses have demonstrated distinct plasma metabolic signatures following COVID-19 vaccination, with significant perturbations in amino acid and lipid profiles correlating with immune response magnitude [26]. These findings suggest that vaccination induces systemic metabolic alterations that may reflect underlying hepatic metabolic changes.

Preclinical animal studies provide detailed insights into this hepatic tropism. In murine models, intramuscular administration of LNP-formulated mRNA vaccines results in substantial hepatic accumulation within hours, with detectable antigen expression in hepatocytes [27,28,29,30]. Consistently, human studies report transient elevations of liver enzymes (ALT, AST) and mild hepatic lipid accumulation, indicating localized metabolic perturbations without overt hepatotoxicity [31,32,33].

Furthermore, comparisons between different LNP formulations, such as (4S)-KEL12 versus SM-102, demonstrate that specific lipid compositions can modulate the degree of hepatic accumulation and transient hepatocellular stress, highlighting the influence of LNP chemistry on biodistribution and metabolic outcomes [34].

Rare clinical cases of autoimmune hepatitis (AIH) following mRNA vaccination highlight the potential for immune-mediated hepatic effects [35]. Reported presentations include elevated liver enzymes, positive autoantibodies (ANA, SMA), and portal lymphocytic infiltration on liver histology [36,37]. Proposed mechanisms involve molecular mimicry, adjuvant effects, epitope spreading, and LNP-mediated antigen presentation, particularly in genetically or metabolically predisposed individuals [38,39]. Nonetheless, population-based studies indicate that such events are exceedingly rare, with no significant increase in AIH incidence observed among millions of vaccinated individuals [35,40,41].

In humans, LNP-mRNA vaccines are associated with transient, mild elevations in liver enzymes reflecting short-term metabolic perturbations [33]. Importantly, these effects are generally self-limiting, with no evidence of sustained liver function impairment or long-term hepatic toxicity in healthy individuals [8,32].

Collectively, these preclinical and clinical data position the liver as a central hub for LNP-mRNA vaccine metabolism and immune interactions. Hepatic accumulation can induce transient metabolic perturbations and, in rare predisposed individuals, trigger autoimmunity, highlighting a plausible role for the liver in systemic metabolic alterations that may influence hematopoietic homeostasis and potentially leukemogenic processes.

### 1.2. Hepato-Hematopoietic Metabolic Crosstalk

The liver-bone marrow metabolic axis represents a critical regulatory network governing hematopoietic homeostasis. The liver supplies essential metabolites for hematopoiesis, including folate cofactors for DNA synthesis, amino acids for protein synthesis, lipids for membrane biogenesis, and iron for hemoglobin production [13,14,16,42]. This metabolic crosstalk is bidirectional, with hematopoietic cells releasing factors that influence hepatic metabolism, including cytokines, growth factors, and metabolic byproducts [43,44,45].

Additionally, hepatic inflammatory responses can generate systemic inflammatory mediators that influence the bone marrow microenvironment [46]. The liver’s role in iron homeostasis is particularly relevant, as hepatic hepcidin production regulates systemic iron distribution and can significantly impact erythropoiesis and overall hematopoietic function [47,48,49].

### 1.3. Metabolic Vulnerabilities in Leukemogenesis

Leukemic cells exhibit characteristic metabolic reprogramming that distinguishes them from normal hematopoietic cells. These alterations include enhanced glycolysis, increased glutaminolysis, dysregulated lipid metabolism, and perturbed one-carbon metabolism [50,51,52]. Pre-leukemic clones, which harbor some but not all genetic alterations required for full malignant transformation, may be particularly vulnerable to metabolic perturbations that provide selective advantages for their expansion [53,54,55].

Recent evidence from our previous study of metabolomic profiling of leukemic bone marrow samples following mRNA vaccination has revealed distinct metabolic signatures, including alterations in glycolysis, pentose phosphate pathway activity, and tryptophan metabolism [2]. While these changes do not establish causality, we demonstrate that vaccination can induce detectable metabolic alterations in the leukemic bone marrow microenvironment.

## 2. Proposed Mechanisms of Hepatic Metabolic Amplification in Leukemogenesis

We propose five interconnected mechanisms through which vaccine-induced hepatic metabolic perturbations may amplify leukemogenic processes in susceptible individuals. These mechanisms operate within the framework of hepato-hematopoietic metabolic crosstalk and may synergistically create a permissive milieu for leukemic transformation or clonal expansion.

### 2.1. Competitive Folate Sequestration and One-Carbon Metabolism Dysregulation

#### 2.1.1. Established Knowledge: Mechanistic Basis

Folate-dependent one-carbon metabolism represents a critical metabolic network supporting nucleotide synthesis, methylation reactions, and redox homeostasis [56,57,58]. The liver serves as the primary site for folate processing, converting dietary folates to their bioactive forms and regulating their systemic distribution [59,60].

Folate deficiency contributes to leukemogenesis through multiple interconnected mechanisms. The deficiency induces DNA damage, promotes chromosomal instability, and compromises DNA repair capacity—collectively creating a mutagenic environment conducive to malignant transformation [61,62,63]. Beyond genomic instability, perturbations in folate-dependent one-carbon metabolism disrupt DNA methylation patterns, thereby altering the epigenetic regulation of proto-oncogenes and tumor suppressor genes [58,61,64,65,66,67].

Pre-leukemic clones exhibit particular vulnerability to folate limitation due to their heightened proliferative demands, which substantially increase cellular folate requirements [58]. However, this metabolic stress may paradoxically confer selective advantages to certain clonal populations. Pre-leukemic clones harboring genetic alterations that enhance folate transport efficiency or optimize folate utilization pathways may demonstrate preferential expansion under folate-restricted conditions. This selective clonal advantage could accelerate the progression from pre-leukemic states to overt leukemic transformation [61,68].

Concurrently, folate deficiency compromises immune surveillance mechanisms, establishing an immunosuppressive microenvironment that facilitates leukemic immune evasion. The impairment of folate-dependent immune cell functions creates permissive conditions for transformed clones to escape immunological detection and elimination, further promoting leukemic progression [58,61,69].

Experimental models have characterized the temporal dynamics and threshold levels of folate perturbation necessary to influence clonal hematopoiesis, providing insights relevant to clinical scenarios. In mice, folate deficiency induces measurable hematopoietic alterations within 35–48 days, indicating that even short-term insufficiency can affect blood cell production [70]. More prolonged folate restriction is required for sustained clonal effects; transplantation studies demonstrate significant reductions in hematopoietic stem/progenitor contributions to B-cell, myeloid, and multipotent populations at seven months post-transplant following dietary folate insufficiency [71]. both facilitating the emergence or expansion of mutant hematopoietic clones over weeks to months [71].

#### 2.1.2. COVID-19 mRNA Vaccines, Spike Protein, and Folate Metabolism

The interaction between COVID-19 mRNA vaccines, the SARS-CoV-2 spike protein, and host folate metabolism is multifaceted and warrants careful consideration [72,73,74]. The mRNA vaccines work by instructing host cells to produce the spike protein, which then triggers an immune response [75]. The administration of mRNA vaccines elicits a robust immune response characterized by rapid proliferation of lymphocytes, including T and B cells [76,77]. This cellular expansion is metabolically demanding, particularly in terms of nucleotide synthesis, which is heavily reliant on adequate folate availability. As highlighted in the initial context, this increased demand for DNA synthesis and cellular replication directly translates to a substantial increase in folate requirements [78]. understanding of this process is further supported by studies demonstrating that folate deficiency can inhibit the proliferation of primary human CD8+ T lymphocytes in vitro, underscoring the critical role of folate in effective adaptive immune responses [79]. Similarly, the proper functioning of natural killer (NK) cells, vital components of the innate immune system involved in viral responses, is also dependent on the folate cycle [80].

Studies on metabolomic alterations induced by SARS-CoV-2 and COVID-19 vaccination have revealed changes in plasma metabolic profiles, with amino acid metabolism and lipid profiles identified as predictive markers of immune response [81,82]. Our previous study specifically investigating the effects of BNT162b2 mRNA COVID-19 vaccine administration on leukemic bone marrow metabolomic profiles observed distinct metabolic changes, including the presence of tetrahydrofolic acid as a notable metabolite [2]. This direct link between vaccine administration and metabolomic differences in bone marrow, particularly involving a key folate derivative, provides strong support for the hypothesis that vaccine-induced immune activation may transiently increase hepatic folate utilization, leading to competitive sequestration and limiting folate availability for bone marrow hematopoietic cells. The liver, being the primary site for folate processing, would be at the forefront of this increased demand during a systemic immune response [13,59,83].

Furthermore, the SARS-CoV-2 virus itself has been shown to remodel host folate and one-carbon metabolism to support its own replication, specifically for de novo purine synthesis required for its genome [72]. This viral manipulation of host metabolism highlights the vulnerability of folate pathways during infection. Interestingly, some research indicates that folic acid might inhibit the binding of SARS-CoV-2 spike proteins, potentially blocking viral entry into cells [84,85]. Conversely, other data suggest that folate supplementation could paradoxically increase viral replication in host cells [86]. This apparent contradiction underscores the complex and context-dependent nature of folate’s role in viral infections and immune responses. The spike protein’s interaction with host cells, whether from the virus or vaccine-induced, appears to directly or indirectly influence folate availability and utilization. For instance, furin inhibitors, which block SARS-CoV-2 spike protein cleavage and suppress virus production, operate in a pathway where folate metabolism plays a role [87,88].

Additionally, SARS-CoV-2 infection has been linked to increased methyl-group requirements and other disturbances of one-carbon metabolism [89]. The potential for folate to bind to the spike glycoprotein on the coronavirus membrane and influence host cell immune response further emphasizes the intricate relationship [85].

## 3. Hypothesis

Vaccine-induced immune activation necessitates rapid lymphocyte proliferation, which substantially increases folate requirements for DNA synthesis and cellular replication [75]. This heightened metabolic activity during immune responses suggests a potential interplay between vaccine administration and folate metabolism. This discussion explores the potential impact of COVID-19 mRNA vaccines, particularly the role of the spike protein, on folate metabolism, drawing upon existing literature and the hypothesis of competitive folate sequestration.

Folate-deficient and excessively supplemented diets altered DNA replication in B-progenitor cells and reduced circulating lymphocyte counts, suggesting a narrow window in which both deficiency and excess compromise hematopoietic function [71]. This timeframe aligns with clinical observations that vaccine-induced immune activation typically peaks within days to weeks [90], implying that transient folate sequestration during this period could modulate hematopoietic stem cell behavior and clonal dynamics. Such effects may be particularly relevant in individuals with marginal baseline folate status, where even modest additional metabolic demands may lower folate availability below the threshold required for effective stem cell maintenance and genomic integrity. These findings collectively suggest that the immune response triggered by COVID-19 mRNA vaccines, and the presence of the spike protein, can significantly influence folate metabolism. This influence can manifest as increased demand due to immune cell proliferation, alterations in related metabolic pathways (like amino acid metabolism), and potentially direct interactions between folate and the spike protein itself. The implications are particularly significant for individuals with pre-existing marginal folate status or genetic polymorphisms affecting folate metabolism, such as methylenetetrahydrofolate reductase (MTHFR) variants, who may be more susceptible to folate imbalances during vaccine-induced immune activation [91].

### 3.1. Hepatic Lipid Processing Overload and Membrane Lipid Dysregulation

#### 3.1.1. Established Knowledge: Mechanistic Basis

Lipid nanoparticles (LNPs), integral to mRNA vaccine formulations, comprise ionizable cationic lipids, phospholipids, and cholesterol [18]. Following systemic administration, these LNPs are predominantly processed by the liver [30]. This preferential hepatic accumulation can transiently overwhelm the liver’s lipid processing machinery, potentially altering systemic lipid homeostasis and membrane lipid composition [6,18,92]. Research indicates that the physicochemical properties of LNPs, including size, charge, and chemical composition, significantly influence their interactions with biological barriers and their biodistribution. For instance, studies have shown that LNP elasticity can impact cellular uptake, with stiffer LNPs exhibiting greater uptake in macrophages and T cells, while softer LNPs tend to accumulate in tumors [18,93,94]. The liver’s role in processing these substantial quantities of lipids from LNPs may transiently disrupt its normal metabolic functions. This disruption could lead to a perturbation in phospholipid metabolism, thereby affecting the composition and distribution of bioactive lipid species. A particularly significant area of concern is sphingolipid metabolism, which produces signaling molecules vital for cell survival, proliferation, and apoptosis [95,96,97]. Recent metabolomic analyses have indeed revealed significant alterations in sphingolipid metabolism after COVID-19 vaccination, with distinct metabolic signatures depending on the vaccine type [26,81]. The intricate dance of lipid metabolism within the liver is a finely tuned process, essential for maintaining systemic lipid homeostasis. The ionizable cationic lipids, such as ALC-0315 and SM-102, are critical for LNP stability and endosomal escape [98]. However, their metabolism within hepatocytes can lead to the generation of various lipid metabolites, some of which may have biological activity. The liver’s capacity to metabolize these novel lipid species, especially when administered in large quantities, is a key determinant of potential overload [99,100]. Phospholipids, like DSPC (1,2-distearoyl-snglycero-3-phosphocholine) and DOPE (1,2-dioleoyl-sn-glycero-3-phosphoethanolamine), are integral to LNP structure and also contribute to the overall lipid load. These phospholipids are typically metabolized through established pathways, but an acute influx can transiently saturate the enzymes involved in their hydrolysis and re-synthesis. This saturation can lead to an accumulation of lysophospholipids or other phospholipid intermediates, which can have detergent-like properties and potentially disrupt cellular membranes or signaling pathways [101,102,103,104]. Cholesterol, another major component of LNPs, is primarily handled by the liver through uptake, esterification, and excretion into bile. An increased influx of cholesterol can upregulate cholesterol synthesis pathways and potentially alter the intracellular cholesterol pools, impacting membrane fluidity and the function of cholesterol-dependent proteins [105,106].

From a quantitative perspective, the lipid mass delivered by a single LNP-based mRNA vaccine dose is modest but non-negligible relative to hepatic lipid metabolism. Each LNP-based mRNA vaccine dose delivers lipids in the milligram range, with ionizable lipids comprising about half of the formulation with cholesterol and phospholipids making up most of the remainder [17]. Although this is minor compared to the several grams of lipids processed daily through lipoprotein secretion, bile acid synthesis, and fatty acid oxidation, the distinction lies in the physicochemical novelty of synthetic lipids. Compounds such as ALC-0315 and SM-102 exhibit slower hepatic clearance compared with endogenous lipids and have limited characterization of their metabolic pathways, which may transiently burden hepatocytes and influence lipid droplet dynamics [100,107].

The concept of “hepatic lipid processing overload” reflects not simple accumulation but activation of stress responses. Inefficient handling of exogenous lipids can trigger endoplasmic reticulum (ER) stress and the unfolded protein response (UPR), while excessive metabolic activity promotes reactive oxygen species generation and oxidative stress. Sustained activation of these pathways may impair hepatocyte function, foster inflammation, and influence systemic metabolic and immune regulation [108,109,110,111,112].

The perturbation of sphingolipid metabolism is a particularly sensitive indicator of metabolic stress. Sphingolipids are not only structural components of membranes but also act as crucial signaling molecules [113,114]. The balance between ceramide (pro-apoptotic) and sphingosine-1-phosphate (pro-survival/proliferative) is tightly regulated [115]. An imbalance, potentially induced by LNP components or the cellular stress response to lipid overload, can shift cells towards apoptosis or uncontrolled proliferation. For example, increased ceramide levels can induce mitochondrial dysfunction and activate stress-activated protein kinases, leading to cell death. Conversely, an increase in sphingosine-1-phosphate can promote cell survival and angiogenesis [116,117,118]. Reported alterations in sphingolipid profiles following vaccination highlight the need for further studies on their long-term consequences for cellular homeostasis and disease susceptibility [26,81].

#### 3.1.2. Leukemogenic Implications

Membrane lipid composition is a critical determinant of cellular signaling, especially through lipid rafts. These specialized membrane microdomains act as platforms for receptor clustering and signal transduction. Any alterations in the composition of membrane lipids can directly influence the activity of key receptors, such as growth factor receptors, cytokine receptors, and adhesion molecules, all of which are crucial regulators of hematopoietic cell proliferation, differentiation, and survival [119,120,121,122,123]. Pre-leukemic clones are often characterized by dysregulated lipid metabolism, exhibiting changes in phospholipid composition that favor enhanced proliferation and survival [124,125].

##### Hypothesis

mRNA vaccine–induced hepatic lipid overload, combined with spike protein–mediated lipid dysregulation, transiently alters systemic lipid metabolism and membrane composition. These changes may disturb lipid raft signaling, modulate sphingolipid balance, and increase susceptibility to ferroptosis and oxidative stress. In hematopoietic cells, such perturbations could enhance survival and proliferation of pre-leukemic clones, facilitating clonal expansion or leukemic progression, particularly in individuals with pre-existing metabolic vulnerabilities.

The observed perturbations in lipid metabolism following mRNA vaccination may be further compounded by the direct metabolic effects of the SARS-CoV-2 Spike protein itself. Recent studies show that the SARS-CoV-2 Spike protein directly disrupts cellular lipid homeostasis, significantly increasing lipid accumulation and dysregulating genes involved in lipid metabolism, autophagy, and ferroptosis. This metabolic dysfunction creates heightened susceptibility to lipotoxicity-induced cell death via ferroptosis. The pathway involves nuclear factor erythroid 2-related factor 2 (Nrf2), which paradoxically contributes to cellular dysfunction despite its typical protective role against oxidative stress. Nrf2 inhibition attenuates Spike protein-induced lipotoxicity, suggesting a maladaptive cellular response under metabolic stress conditions [126,127,128]. These effects are consistently observed in cardiomyocyte-like cell lines, providing a plausible molecular explanation for myocarditis and cardiac complications seen in both COVID-19 patients and vaccine recipients [126,129,130,131]. The convergence of lipid dysregulation and ferroptosis susceptibility in cardiac cells may represent a critical pathway linking Spike protein exposure to cardiovascular pathology. These findings suggest that therapeutic strategies targeting lipid metabolism and oxidative stress pathways may help mitigate complications associated with both natural infection and vaccination.

The potential for vaccine-induced perturbations in systemic lipid homeostasis to exacerbate these pre-existing lipid abnormalities is a significant concern. Such exacerbation could potentially accelerate leukemic transformation or clonal expansion [2]. Dysregulated lipid metabolism, particularly involving sphingolipids and fatty acids, is a recognized hallmark of leukemogenesis, crucial for leukemic cell survival, proliferation, and therapy resistance, thus representing a promising therapeutic target [132,133,134]. Therefore, rigorous investigation into these vaccine-induced metabolic changes and their impact on hematopoietic stem and progenitor cells is warranted.

### 3.2. Cytokine-Mediated Indoleamine 2,3-Dioxygenase Upregulation and Tryptophan Catabolism

#### 3.2.1. Established Knowledge: Mechanistic Basis

Indoleamine 2,3-dioxygenase (IDO) catalyzes the rate-limiting step in tryptophan catabolism through the kynurenine pathway, converting tryptophan to N-formylkynurenine [135]. Hepatic IDO expression is primarily regulated by inflammatory cytokines, particularly interferon-gamma (IFN-γ) [136], which is significantly elevated following vaccination [137]. The liver represents a major site of tryptophan catabolism, influencing systemic tryptophan availability and kynurenine pathway metabolite production [138].

#### 3.2.2. Leukemogenic Implications

Enhanced tryptophan catabolism through the kynurenine pathway generates immunosuppressive metabolites that inhibit T-cell proliferation and function, potentially impairing immune surveillance of pre-leukemic or leukemic cells [139,140,141]. Kynurenine and its downstream metabolites, including 3-hydroxykynurenine and quinolinic acid, can directly affect hematopoietic cell behavior through aryl hydrocarbon receptor (AhR) activation and other signaling pathways [142,143].

Leukemic cells frequently overexpress IDO as an immune evasion mechanism, creating a localized immunosuppressive microenvironment [144]. Additionally, perturbations in tryptophan availability may affect protein synthesis in rapidly proliferating hematopoietic cells, potentially creating selective pressures that favor metabolically adapted pre-leukemic clones.

##### Hypothesis

We propose that vaccine-induced cytokine production, particularly IFN-γ, may upregulate hepatic IDO expression, accelerating tryptophan catabolism and increasing kynurenine pathway metabolite production. This upregulation may be particularly pronounced in individuals with pre-existing inflammatory conditions or genetic variants affecting IDO regulation. Recent evidence from a case study of mast cell activation following mRNA-1273 booster vaccination demonstrated dramatically elevated Th2-biased cytokines, including IL-1Ra, IL-5, IL-6, IL-10, IL-11, CXCL10, and GM-CSF, which may influence tryptophan metabolism through direct and indirect mechanisms [145].

SARS-CoV-2 significantly alters the kynurenine pathway of tryptophan metabolism, crucial for NAD+ production and immune/neuronal modulation [146]. Increased IDO enzyme activity is consistently observed in COVID-19 patients, linking this pathway to disease pathophysiology and progression [147]. Prolonged IDO2 activity is also seen in post-acute SARS-CoV-2 sequelae, impacting protein synthesis and potentially leading to autophagy [148]. Inflammatory cytokines like IFN-γ, IFN-β, and IL-6, elevated during acute COVID-19, upregulate IDO. The spike protein’s interaction with ACE2 may drive this dysregulation [149], as sustained inflammatory cytokine elevation post-infection [150] or vaccination provides a continuous stimulus for IDO upregulation, explaining prolonged tryptophan depletion and kynurenine pathway activation in long COVID and post-vaccination syndromes.

### 3.3. Inflammatory Hepcidin Induction and Iron Homeostasis Disruption

#### 3.3.1. Established Knowledge: Mechanistic Basis

Hepcidin, a liver-derived peptide hormone, serves as the master regulator of systemic iron homeostasis by controlling intestinal iron absorption and macrophage iron recycling [151]. Inflammatory stimuli, particularly interleukin-6 (IL-6), potently induce hepatic hepcidin production through STAT3 signaling [152]. Vaccination typically elicits significant IL-6 production as part of the innate immune response [153], potentially affecting hepcidin levels and iron distribution.

#### 3.3.2. Leukemogenic Implications

Iron plays a critical role in hematopoiesis, serving as an essential cofactor for DNA synthesis, mitochondrial function, and oxygen transport [151]. However, excess iron can promote oxidative stress through Fenton chemistry, generating hydroxyl radicals that damage DNA, proteins, and lipids [154,155]. This iron-induced oxidative stress may contribute to genomic instability and mutagenesis in hematopoietic stem and progenitor cells [155,156,157]. thereby potentially influencing clonal dynamics.

A central regulator of systemic iron homeostasis is hepcidin, which limits iron availability for erythropoiesis through its binding to ferroportin [158]. The magnitude and duration of hepcidin perturbation necessary to exert a biologically meaningful effect on clonal hematopoiesis remain incompletely defined [159].

This interplay between iron metabolism and clonal hematopoiesis is particularly relevant in the context of malignant transformation. Leukemic cells frequently display altered iron metabolism, including enhanced uptake to support their high proliferative demands. Perturbations in systemic iron distribution may therefore provide a selective advantage to pre-leukemic clones with increased iron acquisition capacity. Moreover, experimental evidence indicates that iron overload within the bone marrow microenvironment can impair normal hematopoiesis while simultaneously supporting leukemic cell expansion, potentially accelerating disease progression [156,160].

##### Hypothesis

We hypothesize that vaccine-induced inflammation [161] may transiently increase hepatic hepcidin production [162,163], leading to iron sequestration in hepatocytes and macrophages. This acute phase response may subsequently trigger compensatory mechanisms, including increased intestinal iron absorption and enhanced erythrophagocytosis, potentially leading to iron redistribution and localized iron overload in the bone marrow microenvironment.

Acute inflammation, such as that induced by vaccination or infection, transiently elevates cytokines like IL-6, leading to short-term hepcidin induction and temporary iron redistribution. These transient changes generally have limited impact on hematopoietic function, as iron homeostasis and erythropoiesis rapidly return to baseline once the inflammatory stimulus resolves [164,165].

In contrast, clonal hematopoiesis (CH) is associated with chronic, low-grade inflammation. Mutations in genes such as TET2 or DNMT3A are known to drive aberrant inflammatory signaling, particularly in monocytes and macrophages [166]. This sustained inflammatory milieu may persistently elevate hepcidin levels and disrupt iron homeostasis, creating selective pressures that favor the expansion of pre-leukemic or leukemic clones by altering the bone marrow microenvironment [167,168].

Clinical evidence from an exploratory proteogenomic analysis of the CANTOS trial demonstrates that IL-1β inhibition with canakinumab not only improved hemoglobin levels in patients with CH mutations and anemia, but also suppressed hepcidin, proinflammatory cytokines, and myeloid activation pathways [169]. Although longitudinal hepcidin measurements in CH patients remain limited, these findings indirectly support the concept that chronic inflammatory signaling can sustain hepcidin elevation, restrict iron availability, and impair erythropoiesis.

Overall, this distinction highlights how transient hepcidin elevation during acute inflammation rarely reaches thresholds relevant for clonal selection, whereas sustained induction in the setting of CH may exert biologically meaningful effects on hematopoietic and clonal dynamics.

### 3.4. Hepatic NADPH Demand and Redox Homeostasis Perturbation

#### 3.4.1. Established Knowledge: Mechanistic Basis

Nicotinamide adenine dinucleotide phosphate (NADPH) stands as a pivotal reducing equivalent within cellular metabolism, indispensable for a myriad of critical biological processes. Its multifaceted roles encompass robust antioxidant defense mechanisms, the intricate pathways of lipid biosynthesis, and the crucial detoxification of xenobiotic compounds [170,171,172]. The liver, a central metabolic organ, is a primary site for the de novo synthesis of NADPH, predominantly through the pentose phosphate pathway (PPP) and other ancillary metabolic routes [173,174]. This hepatic capacity for NADPH generation is vital for maintaining cellular homeostasis and responding to metabolic challenges.

#### 3.4.2. Leukemogenic Implications

The maintenance of redox homeostasis is critical for hematopoietic stem cell (HSC) function, governing self-renewal, differentiation, and genomic stability [175,176]. NADPH serves as a key cofactor in antioxidant defense systems [177], and its depletion—may disrupt this balance. Elevated ROS levels due to insufficient NADPH availability can lead to oxidative DNA damage, increasing mutagenesis in HSCs and creating a permissive environment for leukemogenic transformation [178,179,180]. Leukemic cells, already under heightened oxidative stress, rely on adaptive mechanisms to sustain NADPH production and redox equilibrium [175,179].

##### Hypothesis

We propose that vaccine-induced hepatic stress may transiently increase NADPH consumption for antioxidant defense and lipid processing, potentially limiting NADPH availability for other tissues, including the bone marrow. This metabolic competition for reducing equivalents may be particularly significant in individuals with pre-existing redox imbalances or genetic variants affecting NADPH production.

### 3.5. Vaccine-Induced Metabolic Perturbations

The ionizable lipids commonly used in LNP formulations, including ALC-0315 and The polyethylene glycol (PEG), require hepatic processing for clearance. This processing involves cytochrome P450-mediated oxidation, which consumes substantial quantities of NADPH through the cytochrome P450 reductase system. Studies have demonstrated that LNPs containing ALC-0315 at high doses (5 mg/kg) increase markers of liver toxicity (ALT and bile acids), supporting the metabolic burden these lipids place on hepatic processing [100].

Clinical evidence supports this metabolic stress. A multi-country analysis of 87 patients documented liver injury occurring 15 days post-vaccination, with 84% presenting hepatocellular patterns and 57% exhibiting immune-mediated hepatitis features. Spatial proteomics analysis revealed hyperexpanded CD8+ T cell clones with SARS-CoV-2 spike-specific sequences concentrated at portal interfaces, expressing cytotoxic granzymes and tissue-resident markers [181]. These findings demonstrate that vaccine-induced immune responses establish persistent hepatic infiltrates with cytotoxic potential [182].

Additionally, the inflammatory response triggered by vaccine components may activate hepatic macrophages and stellate cells, leading to increased production of reactive oxygen species (ROS) and subsequent depletion of cellular antioxidant reserves [183,184]. This oxidative stress necessitates enhanced NADPH consumption for glutathione regeneration and other antioxidant defense mechanisms [185,186,187,188].

#### 3.5.1. SARS-CoV-2 Spike Protein and NADPH Depletion

Beyond the vaccine-induced metabolic perturbations, the SARS-CoV-2 spike protein itself directly contributes to NADPH demand and potential depletion within host cells [189], the binding of the SARS-CoV-2 spike protein to the ACE2 receptor initiates a cascade that leads to the activation of NADPH oxidases (NOX), particularly NOX2. This activation results in a significant increase in reactive oxygen species (ROS) production [190,191]. The cellular machinery, in an attempt to counteract this surge in oxidative stress, consumes NADPH as a critical cofactor for antioxidant defense systems, such as the regeneration of reduced glutathione (GSH) from oxidized glutathione (GSSG) via glutathione reductase. This direct consumption of NADPH for ROS detoxification, triggered by the spike protein’s interaction with ACE2 and subsequent NOX activation, represents a significant drain on cellular NADPH reserves [192,193].

Spike protein-induced oxidative stress precipitates accelerated NADPH consumption that may exceed cellular biosynthetic capacity, resulting in NADPH depletion and subsequent impairment of redox homeostasis and antioxidant defense mechanisms provides a direct link between the spike protein, and the perturbation of cellular NADPH levels, potentially exacerbating the systemic NADPH limitations discussed in the context of vaccine-induced hepatic stress. The combined effect of these factors could create a more profound and sustained impact on NADPH availability, with potential implications for various physiological processes, including hematopoietic stem cell function and leukemogenesis.

However, systemic NADPH limitations—such as those arising from competitive hepatic consumption post-vaccination—could impair these adaptations, selectively favoring clones with enhanced stress resistance or alternative redox-regulatory pathways [194,195]. This metabolic pressure may contribute to disease progression or therapeutic resistance, underscoring the need to explore how transient NADPH deficits influence leukemogenesis [195].

#### 3.5.2. Testable Predictions of Vaccine-Induced Metabolic Perturbations

**Figure 1:**
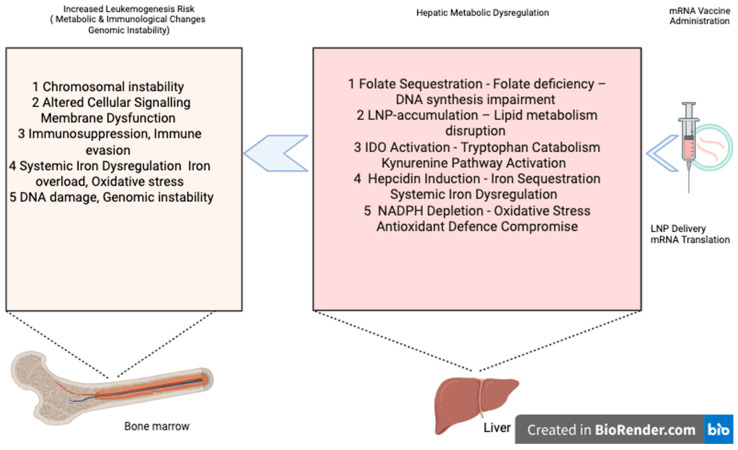
Proposed hepatic metabolic pathways linking mRNA vaccination to leukemogenic susceptibility.

Collectively, these mechanistic pathways suggest that mRNA vaccination may transiently perturb hepatic metabolism through five interconnected processes—folate utilization, lipid processing, tryptophan catabolism, iron regulation, and NADPH/redox balance. These alterations, together with spike protein–mediated effects, could amplify pre-existing leukemogenic vulnerabilities in susceptible individuals (Figure 1). The specific testable predictions and measurable outcomes of these pathways are summarized in Table 1.

## 4. Mechanistic Integration and Synergistic Effects of Our Hypothesis

### 4.1. Temporal Dynamics and Cascade Amplification

The proposed mechanisms operate within distinct temporal frameworks that may create cascading effects amplifying the initial metabolic perturbations. The immediate phase, occurring within hours to days post-vaccination, involves rapid LNP accumulation in hepatocytes and acute inflammatory cytokine release. This phase initiates competitive folate sequestration for immune cell proliferation and increases hepatic NADPH demand for antioxidant defense and lipid processing.

The intermediate phase, spanning days to weeks, encompasses sustained immune activation with persistent cytokine production, leading to prolonged IDO upregulation and hepcidin induction. During this period, the cumulative effects of folate depletion, altered lipid homeostasis, and iron redistribution may create increasingly favorable conditions for pre-leukemic clone expansion. The temporal overlap of these mechanisms may generate synergistic effects that exceed the sum of individual perturbations.

The late phase, extending weeks to months, involves adaptive metabolic responses and potential normalization of acute perturbations. However, in susceptible individuals, the cumulative metabolic stress may have triggered irreversible changes in pre-leukemic clones or bone marrow microenvironment remodeling that persist beyond the resolution of acute effects. This temporal progression suggests that the leukemogenic risk may not be limited to the immediate post-vaccination period but may extend into the recovery phase.

### 4.2. Clinical Implications and Translational Opportunities

The proposed mechanisms suggest that the risk of leukemogenesis following vaccination may be higher in individuals with pre-existing metabolic vulnerabilities or genetic predispositions. For instance, genetic variations affecting folate or iron metabolism could increase susceptibility to folate deficiency or iron-mediated oxidative stress, respectively. Therefore, metabolic biomarker profiling before vaccination could help identify high-risk individuals, allowing for personalized risk assessment and preventive interventions. Routine screening for pre-leukemic conditions like clonal hematopoiesis of indeterminate potential (CHIP) in high-risk populations could also inform vaccination strategies and monitoring protocols [196].

Several preventive interventions are suggested to mitigate leukemogenic risk. Folate supplementation before and after vaccination could prevent folate sequestration and maintain one-carbon metabolism crucial for DNA synthesis and repair [197]. Antioxidant supplementation, particularly N-acetylcysteine, vitamin C, vitamin E, and selenium, may counteract oxidative stress [198]. Iron chelation therapy or dietary modifications could address iron overload. These interventions require careful investigation to ensure efficacy without compromising vaccine immunogenicity.

Potential therapeutic targets for vaccine-associated leukemogenesis include IDO inhibition, which is already in clinical development for cancer immunotherapy [199]. Short-term IDO inhibition post-vaccination could prevent excessive tryptophan catabolism while preserving vaccine immunogenicity. Hepcidin modulation, through agonists to prevent iron redistribution or antagonists to restore normal iron distribution, also presents a therapeutic avenue. Additionally, targeting metabolic pathways to enhance NADPH production or support one-carbon metabolism could maintain redox homeostasis and genomic stability.

Monitoring and surveillance strategies involve tracking specific biomarkers. Folate status, tryptophan metabolism (including kynurenine levels and kynurenine-to-tryptophan ratio), iron metabolism (serum iron, ferritin, transferrin saturation, hepcidin levels, non-transferrin-bound iron, and labile plasma iron), and oxidative stress markers (glutathione ratios, lipid peroxidation products, DNA damage markers) could enable early detection of metabolic perturbations and leukemogenic changes.

### 4.3. Corroborating the Mechanistic Framework: Insights from Clinical Metabolomics

The evolving discourse around mRNA vaccine safety increasingly emphasizes not only immunogenic efficacy but also the broader systemic effects that may accompany lipid nanoparticle (LNP)-mediated delivery systems. Among these, the liver has emerged as a critical organ of interest due to its established tropism for LNPs [6,200]. While the most prominent hepatic effect associated with mRNA vaccination remains a rare and self-limiting autoimmune-like hepatitis [201], a more nuanced perspective suggests that transient hepatic metabolic changes might, under certain circumstances, influence other physiological compartments. In particular, the hypothesis that such hepatic perturbations could act as metabolic amplifiers of pre-existing leukemogenic susceptibility warrants exploration. This integrated analysis aims to contextualize this hypothesis with preliminary clinical findings derived from the metabolomic profiling of leukemic bone marrow samples obtained following BNT162b2 vaccination [2].

A proposed framework describes five mechanistic pathways through which the liver may modulate systemic metabolism with potential implications for hematopoiesis. The first of these involves competitive folate sequestration, whereby vaccine-induced lymphoproliferation increases the demand for folate, potentially depleting hepatic stores and reducing folate availability to the bone marrow [58]. This concept finds preliminary clinical support in Erdogdu et al. [2], who observed lower levels of tetrahydrofolic acid—a biologically active form of folate—in the bone marrow of vaccinated leukemia patients compared to unvaccinated controls Although causality cannot be inferred, this observation may indicate a shift in folate allocation post-vaccination that could subtly affect hematopoietic dynamics, particularly in individuals with proliferative stress or pre-leukemic clonal populations.

A second mechanism, referred to as hepatic lipid processing overload, suggests that the influx and metabolism of LNPs may temporarily disrupt lipid handling in the liver, potentially leading to systemic lipid alterations. In turn, such shifts could affect membrane composition and function in leukemic cells, which are often characterized by altered lipid metabolism [126]. Consistent with this, Erdogdu et al. [2], reported significantly decreased levels of phosphorylcholine—a critical phospholipid component—in bone marrow samples. While the origin of this change remains unclear, it may reflect disturbances in membrane biosynthesis or turnover within the leukemic microenvironment, possibly influenced by systemic lipid perturbations following vaccination

The third hypothesized mechanism focuses on cytokine-mediated modulation of tryptophan metabolism. Vaccine-induced immune activation may elevate interferon-gamma (IFN-γ) levels, thereby stimulating hepatic indoleamine 2,3-dioxygenase (IDO) activity and promoting tryptophan catabolism into immunosuppressive metabolites [139]. In the study by Erdogdu et al. [2], elevated levels of 5-methoxyindoleacetate—a derivative of tryptophan metabolism—were detected in leukemic bone marrow. While this compound is not a direct product of the kynurenine pathway, its accumulation may signify broader alterations in tryptophan handling, raising the possibility that immune-mediated enzymatic pathways such as IDO may modulate the marrow environment in ways that impact leukemic immune escape or survival.

The fourth proposed pathway involves inflammatory regulation of iron homeostasis via hepcidin. Inflammatory stimuli associated with vaccination could potentially upregulate hepatic hepcidin production, leading to functional iron sequestration and reduced availability for erythropoiesis and heme biosynthesis [47]. Erdogdu et al. [2], reported changes in the levels of uroporphyrinogen I/III, intermediates in the heme synthesis pathway, suggesting a possible disruption in iron-dependent metabolic processes within the marrow. Although indirect, this observation may hint at altered iron metabolism in the post-vaccination setting, which could exacerbate oxidative stress or impair hematopoietic efficiency in vulnerable individuals.

Finally, a fifth mechanism centers on NADPH competition and redox imbalance. The hepatic processing of LNPs and management of oxidative stress may increase the local demand for NADPH—a critical cofactor for biosynthetic and antioxidant reactions [173]. If systemic NADPH availability is affected, this may impose additional metabolic strain on bone marrow cells, particularly in leukemic contexts where redox homeostasis is already disrupted. Erdogdu et al. [2] observed increased levels of D-erythrose 4-phosphate and sedoheptulose 1-phosphate, suggesting upregulation of the pentose phosphate pathway (PPP), a major source of NADPH. This metabolic adaptation may reflect an attempt to buffer oxidative stress in the leukemic niche, and further systemic NADPH depletion could potentially favor clones better equipped to withstand such conditions.

Taken together, these five hypothetical mechanisms form a conceptual framework in which transient hepatic responses to mRNA vaccination may exert modest yet biologically relevant influences on leukemic hematopoiesis. Although the clinical observations presented by Erdogdu et al. [2] remain preliminary and correlative in nature, they align with the theoretical pathways proposed here and may serve as a foundation for further hypothesis-driven investigation. Importantly, the systemic metabolic shifts associated with vaccination appear minor relative to the profound changes driven by leukemia itself; however, in individuals with underlying hematological vulnerabilities, even subtle metabolic modulation could have meaningful biological consequences. As such, the potential for hepato-hematopoietic metabolic crosstalk in the context of mRNA vaccination deserves further longitudinal and mechanistic study.

### 4.4. Discussion and Critical Analysis

This hypothesis offers novel contributions by integrating hepatic metabolism as a central mediator, shifting from direct vaccine component effects to a liver-amplified perturbation model. Its multimechanistic approach acknowledges the complexity of metabolic networks, proposing that multiple perturbations synergistically create conditions for leukemic transformation. The temporal framework, recognizing immediate, intermediate, and late-phase effects, provides a nuanced understanding and informs intervention timing. The focus on individual susceptibility aligns with precision medicine, suggesting tailored risk mitigation strategies.

### 4.5. Future Directions

While speculative, the proposed framework generates testable hypotheses that can be addressed through complementary approaches. Preclinical studies using hepatocyte–hematopoietic models and genetically predisposed animal systems can define mechanistic plausibility, while prospective clinical cohorts and biomarker studies may clarify translational relevance. Large-scale epidemiologic analyses integrating genomic and metabolic markers will ultimately determine whether these mechanistic perturbations translate into measurable leukemia risk. Such a tiered research agenda provides a pathway for rigorous evaluation while ensuring that public health priorities surrounding vaccination remain protected.

### 4.6. Limitations

The proposed mechanistic pathways are largely theoretical and require empirical validation. The temporal dynamics may not accurately reflect mRNA vaccine kinetics, and individual variability makes universal timelines difficult. Dose–response relationships are undefined, and the specificity of these mechanisms to mRNA vaccines versus other vaccine types needs clarification through comparative studies.

Alternative explanations for vaccine-associated leukemogenesis include direct genotoxic effects of vaccine components, immune-mediated mechanisms (e.g., molecular mimicry, altered immune surveillance), and the unmasking or acceleration of pre-existing subclinical leukemic processes by vaccine-induced immune activation [202,203,204]. Coincidental associations are also a likely explanation, given the high vaccination rates and background leukemia incidence, necessitating careful epidemiological investigation.

The hypothesis integrates with existing knowledge by building on the role of metabolic reprogramming in cancer, the hepatic tropism of lipid nanoparticles, and the established roles of folate metabolism, tryptophan catabolism, iron homeostasis, and redox regulation in hematopoiesis and leukemogenesis. Its novelty lies in proposing how vaccine-induced perturbations in these pathways synergistically create conditions favorable for leukemogenesis.

While the proposed mechanistic pathways linking mRNA COVID-19 vaccination to leukemia risk are biologically plausible, they remain speculative without direct epidemiological evidence. Notably, large-scale studies have not demonstrated an increased incidence of leukemia following mRNA vaccination. For instance, a comprehensive analysis of cancer mortality rates in Japan during the COVID-19 pandemic found no significant rise in cancer deaths attributable to mRNA vaccines. Furthermore, a review of clinical trials and pharmacovigilance data involving millions of participants reported no increase in cancer risk associated with mRNA vaccines, underscoring the absence of evidence for such a link.

In summary, while the proposed mechanistic models warrant further investigation, current epidemiological and clinical data do not substantiate an increased risk of leukemia following mRNA COVID-19 vaccination. These findings should be acknowledged in the limitations section to provide a balanced perspective.

#### 4.6.1. Clinical Significance for Vaccine Safety and Population Health

Confirmation of this theoretical framework would carry profound consequences for contemporary vaccine safety protocols and public health strategies. The proposed mechanisms suggest implementing enhanced metabolic monitoring systems specifically targeting populations with elevated risk profiles, coupled with the development of comprehensive biomarker panels capable of identifying vulnerable individuals prior to vaccination. This approach aligns with emerging precision medicine paradigms, though successful implementation demands significant advances in genetic screening technologies and metabolic profiling capabilities. The challenge lies in balancing individualized risk assessment with population-level screening efficiency while preserving overall vaccination coverage rates.

Low-risk preventive strategies, including targeted nutritional supplementation and metabolic optimization protocols, represent promising interventions for high-risk cohorts. However, their clinical development necessitates extensive research to ensure these approaches do not compromise vaccine-induced immunogenicity. The metabolic pathways identified through this framework may also yield novel therapeutic targets for leukemia prevention and treatment, focusing on metabolic vulnerabilities independent of vaccination history.

#### 4.6.2. Experimental Framework and Research Priorities

Validating these proposed mechanisms requires a multifaceted research strategy integrating preclinical models, clinical investigations, and methodological innovations. Preclinical validation should leverage murine models with genetic predispositions to hematologic malignancies, complemented by humanized mouse systems incorporating human hepatocytes and hematopoietic cells. In vitro approaches—including primary human hepatocyte cultures and co-culture platforms with hematopoietic progenitors—provide essential tools to dissect cellular crosstalk, metabolic flux, and pathway-specific perturbations, while also enabling high-throughput screening of potential interventions.

Clinical validation demands rigorously designed studies that balance scientific depth with ethical imperatives. Prospective observational cohorts of high-risk individuals followed through vaccination periods could reveal associations between metabolic alterations and hematologic outcomes. Biomarker-oriented studies should emphasize temporal profiling of metabolic and cytokine changes in susceptible populations. Interventional trials exploring preventive strategies—such as folate or antioxidant supplementation—must carefully evaluate both safety and preservation of vaccine immunogenicity.

Key methodological considerations include precise timing of sample collection to capture transient metabolic perturbations, adequate cohort sizes to account for inter-individual variability, and sufficiently long follow-up with appropriate controls to clarify causal links within the multifactorial landscape of leukemogenesis.

Immediate research priorities encompass systematic characterization of vaccine-induced hepatic metabolic responses using advanced metabolomic and multi-omics platforms, combined with computational modeling and innovative imaging techniques. Mechanistic investigations should focus on folate metabolism, tryptophan catabolism, iron homeostasis, and redox regulation in hepatocytes and hematopoietic compartments. Clinical research should prioritize biomarker development to translate mechanistic insights into practical tools while maintaining emphasis on preventive interventions.

Regulatory and policy dimensions must include refined metabolic safety assessments in vaccine approval processes, evidence-based risk communication strategies, and clinical guidelines for identifying vulnerable individuals. Throughout, ethical safeguards are essential to ensure that research deepens mechanistic understanding without undermining vaccination confidence, thereby preserving public health priorities while advancing scientific knowledge.

## 5. Conclusions

This hypothesis proposes a novel mechanistic framework linking mRNA vaccination to leukemogenesis in susceptible individuals, mediated by the liver’s role in amplifying vaccine-induced metabolic perturbations across five interconnected pathways: folate utilization, lipid processing, tryptophan catabolism, iron homeostasis, and redox regulation. Its strength lies in integrating established biological principles with novel insights, recognizing individual susceptibility, and generating testable predictions.

However, it remains theoretical and requires rigorous experimental and clinical validation, acknowledging the complexity and multifactorial nature of leukemogenesis. The potential risks must be weighed against the well-established benefits of vaccination. Clinical implications include personalized risk assessment, preventive interventions, and enhanced monitoring. Research priorities extend to broader questions of metabolic regulation in leukemogenesis and new therapeutic approaches.

Ultimately, this hypothesis calls for increased attention to metabolic factors in vaccine safety and leukemia research, providing a framework for understanding the complex interactions between vaccination, metabolism, and cancer. Validation or refutation through rigorous scientific investigation will improve knowledge of vaccine safety and leukemogenesis, leading to better prevention and treatment strategies. Maintaining public confidence in vaccination programs is paramount, and research must be conducted with scientific rigor and clear communication about the hypothetical nature of proposed mechanisms, aiming to enhance safety through understanding individual risk factors and targeted interventions, rather than discouraging vaccination.

## Figures and Tables

**Table 1:** Testable predictions of vaccine-induced metabolic perturbations.

Mechanism	Testable Prediction	Measurable Outcome
Folate/One-Carbon Metabolism	Transient folate depletion following vaccination	Plasma and bone marrow tetrahydrofolate levels; lymphocyte proliferation and NK cell activity
Hepatic Lipid Processing/Lipid Overload	Alterations in sphingolipid and phospholipid profiles; lipid raft composition	Plasma and liver metabolomics; hepatocyte lipid droplet morphology; ER stress markers
Tryptophan Catabolism/IDO Activation	Increased kynurenine pathway activity mediated by cytokine induction	Kynurenine-to-tryptophan ratio; T-cell proliferation and effector function assays
Iron Homeostasis/Hepcidin Induction	Redistribution of iron between liver, macrophages, and bone marrow	Serum hepcidin, ferritin, transferrin saturation; bone marrow erythropoietic activity
NADPH/Redox Homeostasis	Elevated NADPH consumption and transient oxidative stress	NADPH/NADP+ ratio; reactive oxygen species (ROS) levels; antioxidant enzyme activity
Spike Protein-Specific Effects	Direct modulation of host metabolic pathways, including folate, lipid, and redox metabolism	Hepatocyte or cardiomyocyte lipid accumulation; ferroptotic sensitivity; NADPH utilization
Clinical Susceptibility	Individuals with pre-existing metabolic or genetic vulnerabilities exhibit exaggerated responses	Correlation of metabolomic changes with MTHFR polymorphisms, dyslipidemia, chronic inflammation; hematopoietic clonality

Legend: The table outlines the five proposed mechanisms, their predicted effects, and measurable outcomes that can be empirically validated in clinical and experimental settings.

## Data Availability

The data presented in this study are available in the article.
